# Usefulness of peripherally inserted central catheter port system (PICC-PORT) implantation in the sitting position: a new technique for cases unsuitable for conventional implantation

**DOI:** 10.1007/s11604-022-01317-7

**Published:** 2022-08-09

**Authors:** Akiko Narita, Yumi Takehara, Yuki Maruchi, Nozomu Matsunaga, Shuji Ikeda, Yuichiro Izumi, Toyohiro Ota, Kojiro Suzuki

**Affiliations:** Department of Radiology, Aichi Medical University, 1-1 Yazako Karimata, Nagakute, Aichi 480-1195 Japan

**Keywords:** Central venous port, Venous access, PICC port, Upper arm, Sitting position

## Abstract

**Purpose:**

Totally implantable central venous access port implantation is typically performed in the supine position. However, some patients cannot adopt the supine position due to severe pain and/or dyspnea. The present study evaluated the technical feasibility of peripherally inserted central catheter port system (PICC-PORT) implantation in the sitting position in such cases.

**Materials and methods:**

In the sitting position method, PICC-PORT implantation was performed with the patients seated on a videofluoroscopy chair positioned between the limbs of an angiographic C-arm and the operative upper arm positioned on an arm stand.

From January 2019 to September 2021, eight patients underwent PICC-PORT implantations using this sitting method. We also evaluated 251 consecutive patients with conventional supine position PICC-PORT implantation as controls. Differences in technical success, procedure time and complications were retrospectively assessed between the two groups.

**Results:**

Procedural success rates were 100% in both groups. Median procedure times in the sitting and conventional groups were 42 and 44 min, respectively. No complications were observed in the sitting group. There were no significant differences between the two groups in procedure time (*p* = 0.674) and complications (*p* = 1.000).

**Conclusion:**

Implantation of PICC-PORT in the sitting position is technically feasible and useful.

## Introduction

Totally implantable central venous access ports are devices that are used to gain access to the central vein and are implanted entirely under the skin [1]. They are a closed system consisting of a catheter inserted into the central vein and a reservoir that is accessed through percutaneous puncture. Using these devices, it is possible to easily, safely and repeatedly inject medical agents into the central vein. Therefore, they are useful in patients in whom access to the central vein is required for an extended period, such as for chemotherapy, parenteral nutrition, and/or difficult peripheral venous access [1–3].

Totally implantable central venous access ports are typically inserted via the subclavian, jugular or arm vein [1, 4], and these implantations are performed with the patient in the supine position [5–7]. However, patients who need central venous access are often in poor physical condition, such as terminal cancer patients, and some of them cannot adopt the supine position even for a short time period due to severe pain, dyspnea and/or nausea. We have been performing peripherally inserted central catheter port system (PICC-PORT) implantation in the sitting position in these cases.

The present study evaluated the technical feasibility of PICC-PORT implantation in the sitting position in comparison with implantation in the conventional supine position.

## Materials and methods

### Patients

**Fig. 1:**
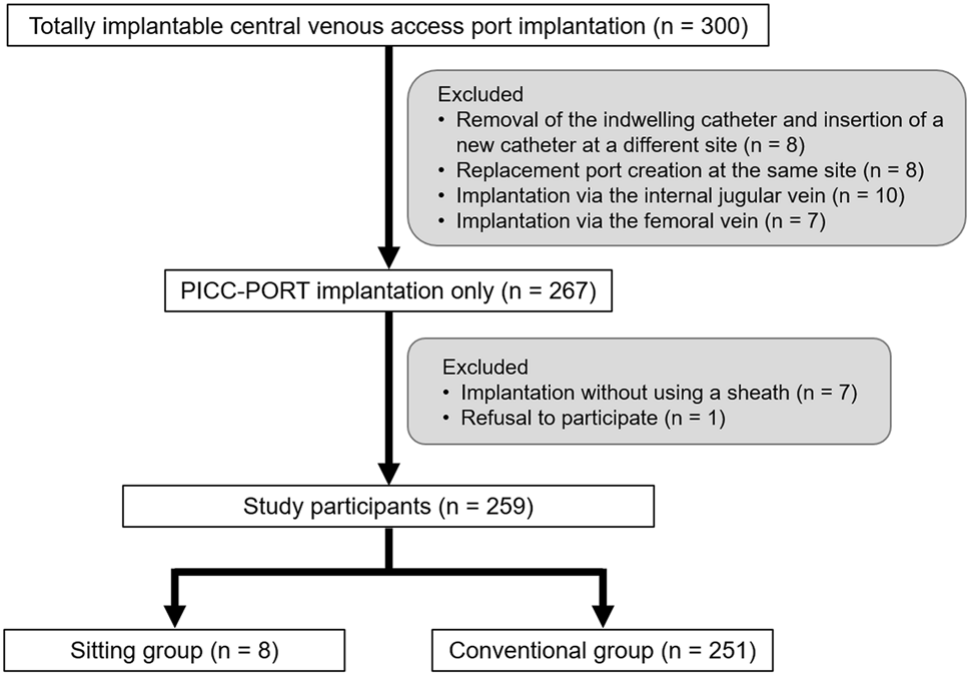
Patient selection

Our institutional review board approved the use of the patients’ clinical data for this study, and the requirement for informed consent was waived because of the retrospective study design. All patients provided written informed consent for totally implantable central venous access port including PICC-PORT implantation. From January 2019 to September 2021, totally implantable central venous access ports were implanted in 300 patients; 33 of these patients were excluded for various reasons such as removal of the indwelling catheter and insertion of a new catheter at a different site (*n* = 8), replacement port creation at the same site (*n* = 8), and implantation via the internal jugular vein (*n* = 10) or femoral vein (*n* = 7). In addition, we excluded eight patients because of implantation without using a sheath (*n* = 7) and refusal to participate (*n* = 1). The remaining 259 patients who underwent PICC-PORT implantation using a peel-away sheath with a check valve were included in this study. Of these, eight patients (3 men and 5 women; median age 69 years; range 37–86 years) who underwent PICC-PORT implantation in the sitting position were included as the sitting group, and 251 patients (103 men and 148 women; median age 72 years; range 18–95 years) who underwent PICC-PORT implantation in the supine position were included as control subjects in this study (Fig. 1).

### Procedure

The procedures were performed using angiographic equipment, Azurion 7 M20 (Philips, Amsterdam, Netherlands) or Artis Q Ceiling (Siemens Healthineers, Munich, Germany). We used a PICC-PORT kit (DewX Eterna, Terumo Corporation, Tokyo, Japan) including a peel-away sheath with a check valve in all cases. All implantations were performed randomly by 11 interventional radiologists with 3 to 31 years’ experience.

### Sitting position method

**Fig. 2:**
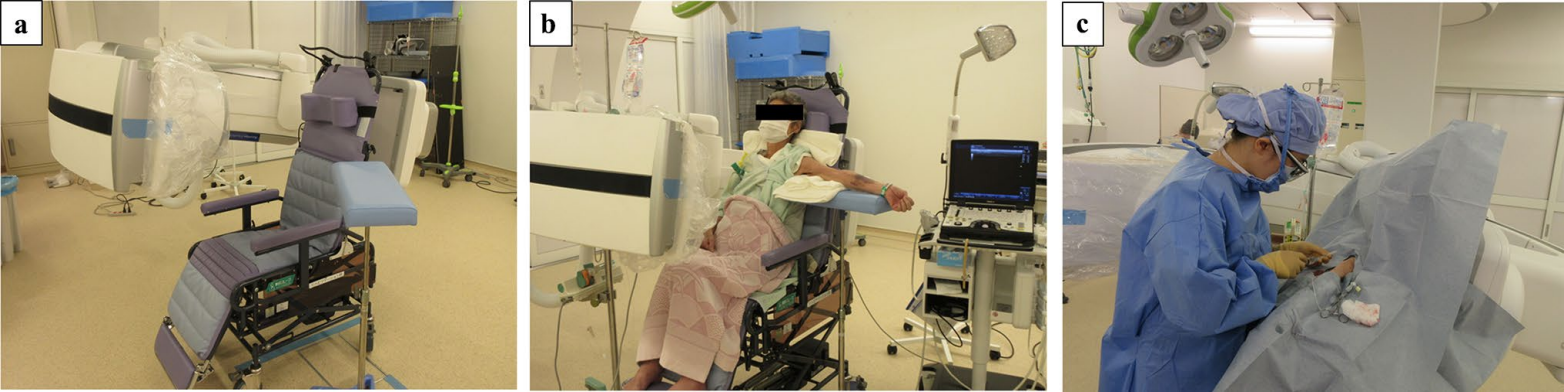
Preparation (**a**, **b**) Equipment used and position of the patient: before the patient entered the operating room, the C-arm of the angiographic equipment was positioned in the anteroposterior direction relative to the videofluoroscopy chair the patient would sit on. With the patient sitting in the chair, the operative upper limb was abducted and the upper arm was placed on an arm stand used for phlebotomy. The seat height was raised so that the patient’s chest was in the imaging range and the adequacy of movement of the C-arm from the chest to the elbow joint was checked before the procedure. **c** Operative procedure

Patients sat on the videofluoroscopy chair (Tomei Brace, Seto, Japan) (Fig. 2a, b). This chair has a radiolucent back section which does not obstruct fluoroscopic visualization during imaging, and also adjustable backrest sections inclination and seat height. The seat height was raised so that the patient’s chest was positioned in the imaging range, and the angle of the seat was adjusted to a position that was comfortable for the patient. The operative upper limb was abducted and the upper arm was positioned in extension on an arm stand used for phlebotomy, to prevent elbow flexion. The C-arm was positioned in the anteroposterior direction relative to the patient (perpendicular to the coronal plane of the body, with the C-arm in the horizontal plane), and the adequacy of movement of the C-arm for visualization of the chest to the elbow joint was confirmed. Then, PICC-PORT implantation was performed (Fig. 2c).

### PICC-PORT implantation

**Fig. 3:**
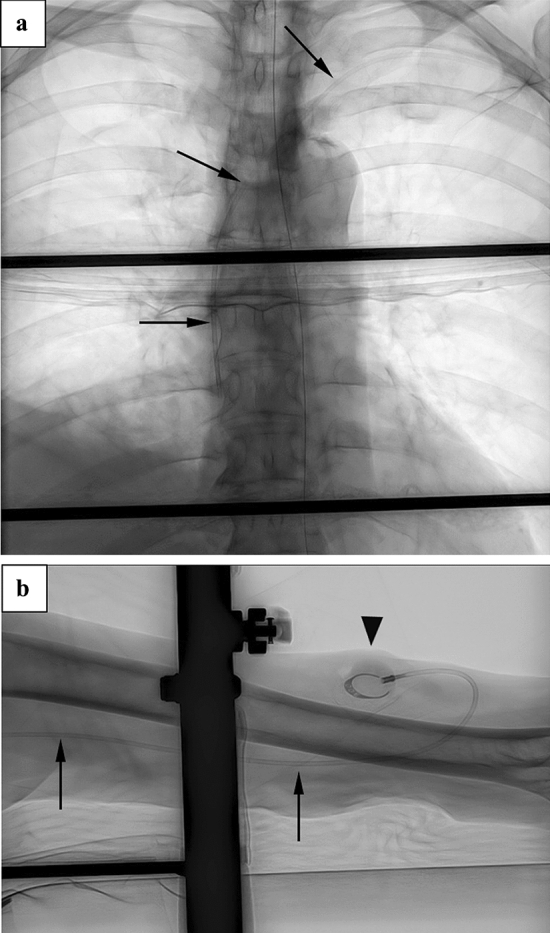
Post-procedure images (**a**) Chest: since the back rest of the videofluoroscopy chair is radiolucent, it allows visualization of the location, movement and course of the guide wire and catheter (black arrow). The catheter tip was positioned at the atriocaval junction. **b** Upper arm: the imaging range included the elbow joint. The port was implanted in the upper arm (black arrow head)

Before the procedure, we checked the patients’ medical history, dominant hand and vessel course pattern using computed tomography, to determine the operative side. The target vein was determined using ultrasonography before the puncture, based on an adequate vascular caliber, distance from the nerve and artery, and patency of the vein. Unless otherwise contraindicated, the basilic vein was the first choice for catheter insertion. After skin disinfection, a tourniquet was applied to the proximal upper arm, and puncture of the vein was performed 5 cm proximal to the elbow joint using real-time ultrasonography under local anesthesia. Inserting a 0.025-inch guide wire slightly to secure blood vessel, a tourniquet was removed. Then, a guide wire was advanced into the superior vena cava under fluoroscopic guidance, and a peel-away sheath with a check valve was introduced. A catheter was inserted along with the guide wire and the catheter tip was adjusted to position it at the atriocaval junction under fluoroscopy, following which the sheath was removed. Next, a subcutaneous pocket was created in line with the puncture in the lateral bicipital groove on the outside of the upper arm. Using a tunneler, a subcutaneous tunnel was created from the puncture site to the pocket, and the catheter was passed through the tunnel. After cutting the catheter to the optimal length, the catheter and port were connected as soon as possible. The port was implanted in the pocket and the wound was subsequently sutured. Finally, the port was accessed percutaneously with a 22-gage non-coring needle, and backflow of blood and smooth injection of saline without occlusion of the catheter or leakage of saline were confirmed. The location of the catheter tip and the course of the catheter were also confirmed fluoroscopically (Fig. 3a, b).

### Data analysis

We retrospectively reviewed the medical records of the patients for information about demographics, underlying disease, and indication for PICC-PORT implantation. In the sitting group, the reason for implantation in the sitting position was also assessed. Regarding procedural data, technical success of the procedure, access site, procedure time, follow-up period, and implantation-related complications were analyzed. Technical success was defined as implantation with the tip positioned in the central vein and adequate catheter function. Procedure time was defined as the time from time-out immediately before the local anesthesia until the last digital angiogram. Implantation-related complications included intraprocedural (during the procedure) and post-procedural (within 30 days after the procedure) complications. We recorded complications that needed therapy. For statistical analysis, Fisher’s exact test and Mann–Whitney test were used. All analysis was performed with SPSS for Windows (version 26.0, SPSS, IBM Corp., Armonk, NY). A p value of 0.05 was considered the limit of significance.

## Results

The characteristics of patients in each group are shown in Table 1. Table 2 demonstrates detailed characteristics of patients in the sitting group. All patients in the sitting group had malignancies, whereas in the conventional group, the leading underlying disease was malignancy in 79.7%, followed by others, such as aspiration pneumonia and decreased activities of daily life (ADL) in 20.3%. No significant differences were observed between the sitting and conventional groups in terms of age, sex and underlying disease. The indications for port implantation in the sitting group were parenteral nutrition and difficult peripheral venous access, while 121 (28.2%) patients in the conventional group required port implantation for chemotherapy. The reasons for implantation in the sitting position were severe back pain (bone metastases, *n* = 3; herniated disc, *n* = 1; strained back, *n* = 1), dyspnea in the supine position (massive pleural effusion, *n* = 2), nausea (malignant gastric stenosis, *n* = 1) and vasovagal syncope (*n* = 1).

**Table 1:** Demographics and clinical characteristics

Patient characteristics	Sitting group (*n* = 8)	Conventional group (*n* = 251)	*P*
Age, year; median (range)	69 (37–86)	72 (18–95)	0.371
Sex (male/female)	3:5	103:148	1.000
Underlying disease, n (%)			0.362
malignancy disease	8 (100.0)	200 (79.7)	
Others	0 (0.0)	51 (20.3)	
Indication for port implantation, n (%)*			
Parenteral nutrition	6 (75.0)	123 (49.0)	0.172
Difficult peripheral venous access	3 (37.5)	132 (52.6)	0.486
Chemotherapy	0 (0.0)	121 (28.2)	0.008

^*^Multiple choices allowed

**Table 2:** Patient in sitting group characteristics and treatment details

Case	Age/sex	Underlying disease	Indication for port implantation	Reason for implantation in the sitting position	Access site	Procedure time (minutes)	Complication
1	37/F	Cervical cancer	Parenteral nutrition, Difficult peripheral venous access	Back pain	Left basilic vein	28	No
2	37/F	Cervical cancer	Difficult peripheral venous access	Back pain, Dyspnea	Left brachial vein	44	No
3	65/M	Ureteral cancer	Parenteral nutrition	Vasovagal syncope	Right brachial vein	37	No
4	73/F	Lung cancer	Parenteral nutrition	Back pain	Left basilic vein	37	No
5	50/F	Endometrial cancer	Parenteral nutrition	Dyspnea	Left basilic vein	42	No
6	82/F	Endometrial cancer	Parenteral nutrition	Back pain	Left basilic vein	79	No
7	86/M	Esophageal cancer	Difficult peripheral venous access	Back pain	Right basilic vein	53	No
8	73/M	Gastric cancer	Parenteral nutrition	Nausea, Vomiting	Left brachial vein	38	No

Table 3 shows the procedural characteristics of each group. Procedural success rates in both groups were 100%. Median procedure times in the sitting and conventional groups were 42 min (range 28–79) and 44 min (range 17–130), respectively. There was no significant difference in procedure time. The median follow-up periods in the sitting and conventional groups were 24.5 days (range 6–85) and 32 days (range 0–1087), which was a statistically significant difference (*p* = 0.031). No intraprocedural or post-procedural complications were observed in the sitting group, while one intraprocedural complication (vagal response, *n* = 1) and four post-procedural complications (small amount of bleeding, *n* = 1; hematoma due to restarting anticoagulation, *n* = 1; wound dehiscence, *n* = 2) were observed in the conventional group. Arterial injury, nerve injury and air embolism were not observed in this case series. There was no significant difference in implantation-related complications between the two groups.

**Table 3:** Procedural characteristics

Patient characteristics	Sitting group (*n* = 8)	Conventional group (*n* = 251)	*P*
Primary success rate (%)	100	100	–
Procedure time, minutes; median (range)	42 (28–79)	44 (17–130)	0.674
Follow-up period, days, median (range)	24.5 (6–85)	75 (0–1087)	0.031
Intraprocedural complications, n (%)			
Arterial injury	0 (0.0)	0 (0.0)	–
Nerve injury	0 (0.0)	0 (0.0)	–
Air embolism	0 (0.0)	0 (0.0)	–
Vagal response	0 (0.0)	1 (0.3)	1.000
Total	0 (0.0)	1 (0.3)	1.000
Postprocedural complications, n (%)			
Hematoma/Bleeding	0 (0.0)	2 (0.8)	1.000
Wound dehiscence	0 (0.0)	2 (0.8)	1.000
Total	0 (0.0)	4 (1.6)	1.000

## Discussion

Totally implantable central venous access ports enable easy, safe and repeated venous access. They are usually implanted with the patient in the supine position [5–7]. Here, we described a technique of PICC-PORT implantation that is performed with the patient in the sitting position. To the best of our knowledge, this method has not been previously described in the literature.

In the present study, although all patients in the sitting group had malignancies, the PICC-PORT were not implanted for chemotherapy, but for parenteral nutrition and difficult peripheral venous access. In addition, most of the reasons for implantation in the sitting position were related to the malignancies and their follow-up periods were short. These observations suggest that the patients in the sitting group all suffered from terminal cancers. The number of implantations performed has recently been increasing due to increased aging of the population and increase in the number of cancer patients [8]. Accordingly, in the future, the number of patients who need central venous access but cannot adopt the supine position might increase. Our sitting method might expand the indications of port implantation for patients in whom the conventional technique cannot be performed.

In this study, the procedural success rate was 100% in the sitting group, and median procedure times were not significantly different between the sitting and conventional groups. In addition, no implantation-related complications were observed in the sitting group and all implantations were performed safely. However, totally implantable central venous access port implantation carries the risk of severe procedural complications, such as arterial injury, nerve injury, and air embolism [1]. In particular, air embolism, although rare, is potentially fatal [9, 10]. Entrance of air into the venous system is caused by (1) open access between the air and venous system and (2) venous pressure being below atmospheric pressure [11].

Serious air embolisms have been reported in rare cases of central venous access via the internal jugular or subclavian vein. It mainly occurs when a catheter is inserted through a peel-away sheath [9]. On the other hand, to the best of our knowledge, there is no literature reporting air embolism in PICC-PORT implantation. Mitsuda et al. discussed that successful placement of a peripherally inserted central catheter in the sitting position could be because venous pressure at the insertion site in a peripheral vein is higher than atmospheric pressure [12]. In our sitting method as well, the approach is via a peripheral vein, and a tourniquet, guidewire and check valve sheath are also used. Therefore, it is unlikely that a large amount of air enters venous system during catheter insertion. Blood pressure is related to gravity and the venous pressure is decreased by 0.77 mmHg for each centimeter above the right atrium in the standing position [13]. Thus, the pressure in the superior vena cava of sitting position is assumed to be lower than that of supine position. Wysoki et al. also reported that 40% of patients underwent central venous catheter placement had negative central venous pressures [14]. Implantation in the sitting position may raise the risk of air embolism while connecting the catheter to a port, because both distal and proximal ends of the catheter are open in that time.

Our study has a few limitations. First, there were a small number of patients. Second, this was a single center, retrospective study. Finally, we could not evaluate the long-term outcomes and complications of PICC-PORT implantation because most patients in the sitting group had terminal cancers and their follow-up periods were short. Further studies with a larger number of cases and under various conditions are needed to investigate the safety of this procedure.

In conclusion, implantation of PICC-PORT in the sitting position is a technically feasible method for patients who cannot adopt the supine position. This technique might expand the indications of totally implantable central venous access port implantation.
